# Patterns of Falls and Fractures Among Older Adults Undergoing Rehabilitation After Fall-Related Fractures: A Retrospective Study in Croatia

**DOI:** 10.3390/geriatrics11050139

**Published:** 2026-10-02

**Authors:** Ivana Herak, Tina Košanski, Valentina Vincek, Mateja Križaj Grabant, Zoran Žeželj, Marijana Neuberg

**Affiliations:** Department of Nursing, University North, Jurja Križanića 31b, 42000 Varaždin, Croatia; ticikac@unin.hr (T.K.); vavincek@unin.hr (V.V.); mkrizaj@unin.hr (M.K.G.); zozezelj@unin.hr (Z.Ž.); mneuberg@unin.hr (M.N.)

**Keywords:** accidental falls, aged, hip fractures, osteoporosis, chronic disease, rehabilitation, fall prevention, gerontological nursing

## Abstract

**Background/Objectives:** The injury patterns of older patients who reach rehabilitation after a fall-related fracture are rarely described. We aimed to describe these patients and to examine how age, sex, recorded chronic conditions, osteoporosis and previous fractures relate to the mechanism of injury and the fracture site. **Methods:** In this retrospective observational study, the records of 255 patients aged 65 years and over who entered a rehabilitation programme at a Croatian rehabilitation hospital after a fall-related fracture between 1 January 2024 and 31 December 2025 were extracted from the hospital information system and analysed using the chi-square test, the Mann–Whitney U test, the Kruskal–Wallis test and Spearman rank correlation. **Results:** Women accounted for 74.9% of the sample and hip fracture was the most frequent site (52.9%). Ground-level falls caused 78.8% of fractures overall, 95.2% of those in patients aged 85 years and over, and 86.7% of hip fractures; the mechanism of injury differed by sex, age group and fracture site (all *p* ≤ 0.005). Cardiovascular disease was recorded in 78.0% of patients but osteoporosis in only 19.2%. Both osteoporosis (*p* = 0.041) and having two or more recorded chronic conditions (*p* = 0.012) were associated with the mechanism of injury, but not with the fracture site. **Conclusions:** Hip fractures and ground-level falls predominated, particularly in the oldest patients. The findings support systematic nursing assessment of fall circumstances, chronic disease burden, bone health and fracture history after a fall.

## 1. Introduction

Falls are common in older age and are associated with injury, loss of functional independence, reduced quality of life and increased demand for health and social care. A European systematic review and meta-analysis published in 2025 estimated that approximately 30% of community-dwelling adults aged 65 years and over experience at least one fall per year, with no clear decline in prevalence across the decades studied [1]. World guidelines for falls prevention and management therefore emphasise early identification of people at increased risk and individually tailored, multicomponent preventive interventions [2].

A fall in older age is rarely attributable to a single cause. Gait and balance impairment, reduced muscle strength, chronic disease, cardiovascular disorders, orthostatic hypotension, cognitive difficulties, medication, vision and hearing impairment, functional limitations and environmental factors may all contribute [2]. The role of health professionals is not only to establish whether a person has fallen, but to understand the circumstances of the fall and the factors that can be modified. This approach is particularly important after a fall that has resulted in serious injury.

Fractures are among the most severe consequences of falls in older adults. Croatian data from Split-Dalmatia County for the period 2020–2024 showed that people aged 65 years and over accounted for more than half of all fall-related hospitalisations, that women were more frequently affected than men, and that hip and femoral injuries were the most common [3]. These findings confirm that falls and their consequences represent a relevant problem within the Croatian health care system as well.

Hip fracture occupies a particular position because of its consequences for mobility, independence and rehabilitation needs. A 2024 systematic review showed that hip fractures in older adults frequently result from falls from standing height or approximately that level, often indoors, and with features suggesting a reduced capacity for protective responses during the fall [4]. A large study of more than 170,000 older adults presenting to emergency services demonstrated a clear increase in the proportion of hip fractures with advancing age, with the highest proportion in those aged 85 years and over [5].

Health status further shapes the vulnerability of an older person. Cardiovascular disorders are associated with an increased risk of falling, and current guidelines recommend cardiovascular assessment as part of a comprehensive risk assessment [2,6]. Cognitive difficulties are also associated with falls; a recent meta-analysis reported that particular aspects of attention, executive function and working memory were related to a higher incidence of falls [7]. In a Norwegian registry study of 9525 people assessed for cognitive symptoms, the annual fall incidence was 39.6% and reduced gait speed, impaired activities of daily living and the use of a walking aid were independent predictors of falling [8]. The coexistence of multiple chronic diseases is common in the older population, but its reported prevalence varies substantially between studies depending on the definition used and the number of conditions monitored [9].

Bone health is central to understanding the consequences of a fall. Osteoporosis increases susceptibility to fracture after low-energy trauma, and a previous fracture is a well-established indicator of increased risk of subsequent fracture [10,11]. However, data drawn from routine clinical documentation may not fully reflect the true frequency of osteoporosis. Recent studies and professional position statements highlight a persistent gap in the diagnosis, documentation and treatment of osteoporosis after fragility fractures [12,13].

For nursing practice, it is important that many fall-related factors can be identified during routine contact with an older person. Assessment of previous falls, mobility, chronic disease, medication, cognitive and functional status, fear of falling and home conditions can guide subsequent actions and the involvement of other team members. Evidence syntheses prepared for the US Preventive Services Task Force indicate that exercise interventions show the most consistent effect on reducing falls and fall-related injuries, while multifactorial interventions reduce the rate of falls with less consistent results across other outcomes [14].

The UNIN Fall Protect project of the Department of Nursing, University North, is directed towards the development of a preventive approach to falls in older adults. As one of its first steps, a retrospective analysis was conducted of people admitted to a rehabilitation programme after a fall-related fracture. This design does not allow the identification of predictors of falls in the general population, but it does permit a detailed description of people in whom a fall resulted in a fracture, the circumstances of injury, and the associations between available demographic and clinical characteristics and the resulting fracture.

### Aim of the Study

The aim of the study was to describe the demographic and clinical characteristics of adults aged 65 years and over who sustained a fracture after a fall, and to examine the associations of age, sex, recorded chronic conditions, osteoporosis and previous fractures with the mechanism of injury and the fracture site. Treatment modality, time to the start of rehabilitation and duration of rehabilitation were analysed additionally. The results were considered as a basis for further planning of preventive activities within the UNIN Fall Protect project.

## 2. Materials and Methods

### 2.1. Study Design and Setting

A retrospective observational study was conducted within the UNIN Fall Protect project of the Department of Nursing, University North. Data were analysed for adults aged 65 years and over who were admitted to a rehabilitation programme following a fall-related fracture between 1 January 2024 and 31 December 2025. The study was carried out at the Special Hospital for Medical Rehabilitation Varaždinske Toplice, Croatia, and all data were extracted from the hospital information system. The hospital is located in Varaždin County but serves a broad referral population from across Croatia. Its institutional profile reports a nominal capacity of 923 beds across five interconnected facilities. During part of the study period, the Minerva facility (404 beds) was unavailable because of reconstruction and renovation, temporarily reducing the hospital’s available rehabilitation capacity. The manuscript was prepared in accordance with the STROBE reporting guideline for observational studies [15]; the completed checklist and the participant selection flow diagram are provided as Supplementary Materials (Table S1 and Figure S1).

### 2.2. Participants

During the predefined observation period, 400 patients aged 65 years and over were admitted to a rehabilitation programme at the study hospital and were assessed for eligibility. Inclusion criteria were age 65 years or over, a documented fracture sustained as a consequence of a fall, and admission to a rehabilitation program during the observation period. A total of 145 patients were excluded because rehabilitation had been indicated for a diagnosis other than a fall-related fracture, leaving 255 participants in the final analysis. All consecutive records meeting the inclusion criteria were included; no further exclusion criteria were applied after the database had been assembled. No a priori sample size calculation was performed because this was a retrospective study with a fixed observation period; the final sample therefore comprised all eligible patients treated during that period. The selection process is presented in Figure S1.

Age was recorded in the source database in three groups: 65–74 years, 75–84 years and 85 years or over. Exact chronological age was not available, and age was therefore not analysed as a continuous variable.

### 2.3. Variables

The following variables were analysed: sex, age group, mechanism of injury, fracture site, treatment modality, type of rehabilitation, time from fracture to the start of rehabilitation, total duration of rehabilitation, mode of discharge, osteoporosis, cardiovascular disease, diabetes mellitus, dementia or cognitive impairment, and previous fractures.

The mechanism of injury was classified as a ground-level fall or a fall from a height. A ground-level fall was defined as a fall from standing height or lower, occurring on the same level as the surface on which the person was standing, walking, standing up or turning. A fall from a height was defined as a fall from a level above the standing surface, including falls from a bed, chair, stairs, ladder or other elevated surface.

Fracture sites comprised hip fracture (S72.0–S72.2), femoral fracture outside the hip region (S72.3–S72.9), lower leg fracture (S82.–), vertebral fracture (S22.–, S32.0) and pelvic fracture (S32.1–S32.5). Only vertebral fractures of the thoracic spine were included in the S22.– category; rib and sternal fractures coded within the same block were not included.

Four health conditions–osteoporosis, cardiovascular disease, diabetes mellitus, and dementia or cognitive impairment–were analysed individually as present or absent. Dementia or cognitive impairment was considered present when it was documented in the medical history contained in the existing hospital record at admission to the rehabilitation programme. No additional cognitive screening or diagnostic assessment was performed specifically for this study. Because the database did not contain a complete list of chronic diseases, the summary variable was not interpreted as overall multimorbidity. The number of recorded conditions (0–4) was retained for descriptive reporting; for inferential comparisons of condition burden, participants were grouped as having 0–1 or ≥2 recorded conditions to avoid sparse cells.

Previous fracture history was analysed as a dichotomous variable (yes/no) for inferential comparisons because the higher-count category in the original recording scheme was sparse. Data on previous fractures were available for 254 participants.

### 2.4. Statistical Analysis

Demographic and clinical characteristics were presented using descriptive statistics. Continuous variables were presented as median, range and interquartile range because of the non-normal distribution of the data. Normality of distribution was assessed using the D’Agostino–Pearson test. Categorical variables were analysed using the chi-square test when expected cell frequencies were adequate. When sparse cells violated chi-square assumptions, Fisher’s exact test was used for 2 × 2 tables and the Fisher–Freeman–Halton exact test for larger contingency tables. For the comparison of treatment modality across fracture sites, vertebral fractures were excluded because their usual treatment pathway differs from that of the other fracture groups; owing to sparse expected counts in the resulting 4 × 2 table, the Fisher–Freeman–Halton exact test was used. The Mann–Whitney U test was used to compare continuous variables between two groups, and the Kruskal–Wallis test between three or more groups. The association between continuous variables was assessed using Spearman rank correlation.

Because only two participants received outpatient rehabilitation, comparisons between outpatient and inpatient rehabilitation are reported descriptively only and were not subjected to inferential testing.

All original statistical analyses were performed using MedCalc version 20.305 (MedCalc Software Ltd., Ostend, Belgium). Additional exact tests introduced during peer-review verification were calculated by exact conditional enumeration of the relevant contingency tables. A value of *p* < 0.05 was considered statistically significant.

### 2.5. Ethical Considerations

The study was conducted within the UNIN Fall Protect project with the prior approval of the Ethics Committee of the Special Hospital for Medical Rehabilitation Varaždinske Toplice (approval number 01-724/2-2026, 28 May 2026). The approval covered the retrospective analysis of data recorded in the hospital information system between 1 January 2024 and 31 December 2025. All data used for the study were anonymised, and no additional intervention was performed for the purposes of this research.

## 3. Results

### 3.1. Participant Characteristics and Distribution of Fractures

The study included 255 participants, of whom 191 (74.9%) were women and 64 (25.1%) were men. Most participants belonged to the 75–84 age group (*n* = 118; 46.3%), followed by the 65–74 age group (*n* = 95; 37.3%), while 42 participants (16.5%) were aged 85 years or over. The distribution by sex differed significantly across age groups (*p* = 0.008). Women were more numerous in all age groups and accounted for 92.9% of participants aged 85 years and over.

The most common fracture site was the hip, recorded in 135 participants (52.9%). Lower leg fracture was present in 38 participants (14.9%), femoral fracture outside the hip region in 35 (13.7%), vertebral fracture in 33 (12.9%) and pelvic fracture in 14 participants (5.5%). Fracture site differed significantly by sex (*p* < 0.001), whereas the association between fracture site and age group was not statistically significant (*p* = 0.188). Basic characteristics of the sample are shown in Table 1.

### 3.2. Mechanism of Injury and Treatment Modality

A ground-level fall was the most frequent mechanism of injury and was recorded in 201 participants (78.8%), while 54 fractures (21.2%) resulted from a fall from a height. As shown in Table 2, the mechanism of injury differed significantly by sex (*p* < 0.001): a fall from a height was recorded in 35.9% of men and 16.2% of women. A significant association was also found between age group and mechanism of injury (*p* = 0.005). In the group aged 85 years and over, 40 of 42 participants (95.2%) sustained their fracture after a ground-level fall.

The mechanism of injury also differed by fracture site (*p* < 0.001). A ground-level fall was the cause of 117 of 135 hip fractures (86.7%) and 32 of 35 femoral fractures outside the hip region (91.4%). Falls from a height were proportionally more frequent among lower leg, vertebral and pelvic fractures. The distribution of the mechanism of injury according to selected characteristics is presented in Table 2.

Most participants were treated surgically (*n* = 228; 89.4%), while 27 participants (10.6%) were treated conservatively. Treatment modality was not associated with sex (*p* = 0.567), age group (*p* = 0.617) or mechanism of injury (*p* = 0.103). For the comparison of treatment modality by fracture site, vertebral fractures were excluded, leaving 222 participants. Treatment modality remained significantly associated with fracture site (Fisher–Freeman–Halton exact test, *p* = 0.002). Surgical treatment was used in 95.6% of hip fractures, 94.3% of femoral fractures outside the hip region, 86.8% of lower leg fractures and 64.3% of pelvic fractures.

Almost all participants were included in inpatient rehabilitation (*n* = 253; 99.2%), while only two participants were rehabilitated on an outpatient basis. Because of this extreme imbalance, the type of rehabilitation was not analysed inferentially.

### 3.3. Time to the Start and Duration of Rehabilitation

The median time from fracture to the start of the recorded rehabilitation episode was 110 days, with a range of 5 to 672 days and an interquartile range of 94 days. Time to the start of rehabilitation did not differ significantly by age group (*p* = 0.473) or sex (*p* = 0.345), but differed significantly by fracture site (*p* < 0.001). The shortest median was recorded for hip fractures (97 days), followed by femoral fractures outside the hip region (103 days) and vertebral fractures (113 days). The longest time to the start of rehabilitation was recorded for participants with a lower leg fracture, with a median of 150 days.

Time from fracture to the start of rehabilitation also differed by treatment modality. Conservatively treated participants began rehabilitation later than surgically treated participants, with a median of 148 versus 104 days (*p* < 0.001).

The median total duration of rehabilitation was 21 days, with a range of 4 to 51 days. Duration of rehabilitation did not differ significantly by age group, sex, fracture site or treatment modality. Descriptively, the two participants who received outpatient rehabilitation had a shorter median duration (12 days) than participants in inpatient rehabilitation (21 days); no inferential comparison was performed. Data on time to the start and duration of rehabilitation are shown in Table 3.

A weak negative correlation was found between the time from fracture to the start of rehabilitation and the total duration of rehabilitation (Spearman’s rho = −0.231; 95% CI −0.344 to −0.110; *p* < 0.001).

### 3.4. Recorded Chronic Conditions

At least one of the four recorded chronic conditions was present in 218 participants (85.5%). One condition was recorded in 124 participants (48.6%), two in 79 (31.0%) and three in 14 (5.5%), while one participant had all four recorded conditions. Thirty-seven participants (14.5%) had none of the recorded conditions. In total, 36.9% of participants had two or more recorded chronic conditions.

For inferential comparisons, recorded condition burden was analysed as 0–1 versus ≥2 conditions. Having two or more recorded conditions was not significantly associated with sex (*p* = 0.438), treatment modality (*p* = 0.984), fracture site (*p* = 0.608) or age group (*p* = 0.271). Rehabilitation duration also did not differ between participants with 0–1 and ≥2 recorded conditions (*p* = 0.389).

Having two or more recorded chronic conditions was significantly associated with the mechanism of injury (*p* = 0.012). Two or more recorded conditions were present in 40.8% of participants who sustained their fracture in a ground-level fall, compared with 22.2% of those who fell from a height.

Cardiovascular disease was the most frequently recorded condition, present in 199 participants (78.0%). Diabetes mellitus was recorded in 66 participants (25.9%), osteoporosis in 49 (19.2%) and dementia or cognitive impairment in 14 participants (5.5%). None of these conditions was significantly associated with fracture site (Table 4).

Osteoporosis was significantly associated with the mechanism of injury (*p* = 0.041). Of the 49 participants with recorded osteoporosis, 44 (89.8%) sustained their fracture in a ground-level fall. Among participants without recorded osteoporosis, the proportion of ground-level falls was 76.6%. No significant association with the mechanism of injury was found for cardiovascular disease (*p* = 0.126), diabetes mellitus (*p* = 0.082) or dementia or cognitive impairment (Fisher’s exact test, *p* = 0.740).

### 3.5. Previous Fractures

Data on previous fractures were available for 254 participants. At least one previous fracture was recorded in 65 participants (25.6%), while 189 participants (74.4%) had no recorded previous fracture.

Previous fracture history was not significantly associated with sex (*p* = 0.386), age group (*p* = 0.301), osteoporosis (*p* = 0.752) or having two or more recorded chronic conditions (*p* = 0.339).

### 3.6. Characteristics of Participants with Hip Fracture

A hip fracture was present in 135 of 255 participants (52.9%). The frequency of hip fracture did not differ significantly by sex (*p* = 0.262), presence of osteoporosis (*p* = 0.740), dementia or cognitive impairment (*p* = 0.383), diabetes mellitus (*p* = 0.762) or higher recorded condition burden (≥2 versus 0–1 conditions; *p* = 0.271).

The proportion of hip fractures increased across the age groups studied: 43 of 95 participants aged 65–74 years (45.3%), 64 of 118 participants aged 75–84 years (54.2%) and 28 of 42 participants aged 85 years and over (66.7%). This difference did not reach statistical significance (*p* = 0.064). Hip fracture was somewhat more frequent among participants with cardiovascular disease than among those without (55.8% versus 42.9%), but this association was not statistically significant either (*p* = 0.088).

A significant association was found between hip fracture and the mechanism of injury (*p* = 0.001). Among participants with a hip fracture, 117 of 135 (86.7%) were injured in a ground-level fall, whereas among participants with fractures at other sites the proportion of ground-level falls was 70.0%.

Participants with a hip fracture started the recorded rehabilitation episode earlier than participants with fractures at other sites. The median time from fracture to the start of rehabilitation was 97 days for hip fractures and 125 days for other fractures (*p* = 0.001). The total duration of rehabilitation did not differ between the groups, with a median of 21 days in both (*p* = 0.766). These data are presented in Table 5.

## 4. Discussion

This retrospective study analysed the characteristics of 255 adults aged 65 years and over who entered a rehabilitation programme after a fall-related fracture. Three findings stand out. First, hip fracture was the most frequent fracture site and accounted for more than half of all recorded fractures. Second, ground-level falls were the dominant mechanism of injury, particularly in the oldest age group and among participants with hip fracture. Third, most participants had at least one of the four recorded chronic conditions, and both having two or more recorded conditions and recorded osteoporosis were associated with the mechanism of injury, but not with fracture site or hip-fracture status.

Women accounted for almost three quarters of the sample, and their share was particularly high in the group aged 85 years and over. This distribution corresponds to the findings of a recent Croatian study of fall-related hospitalisations in Split-Dalmatia County, in which women represented 69.8% of hospitalised people aged 65 years and over and the hospitalisation rate was higher in women than in men [3]. In our sample, fracture site was associated with sex, but hip fracture itself, when analysed against all other sites, showed no statistically significant association with sex. This suggests that sex differences should not be reduced to the question of hip fracture alone, but should be considered as part of a broader pattern of injury.

Hip fracture was recorded in 52.9% of participants. This result is comparable with Croatian data indicating that hip and femoral injuries accounted for approximately half of fall-related hospitalisations in the older population, although it should be noted that the Croatian registry category combines hip and femoral injuries and is therefore broader than the hip fracture definition applied here [3]. In our study, the proportion of hip fractures increased from 45.3% in the 65–74 age group to 66.7% in the group aged 85 years and over, although the overall difference did not reach statistical significance. A large Korean study of more than 170,000 people aged 65 years and over demonstrated a marked increase in the likelihood of hip fracture with age, with the highest frequency in the group aged 85 years and over [5]. Our result points in the same direction, but the sample size, particularly of the oldest group, limits the strength of the conclusions that can be drawn.

The predominance of ground-level falls is particularly important for prevention. Such falls accounted for 78.8% of all injuries, 95.2% of injuries in people aged 85 years and over, and 86.7% of hip fractures. The systematic review by Lim and colleagues showed that hip fractures in older adults frequently occur after a fall from standing height or a similar level, and that the reported circumstances often reflect reduced physical reserve and a weaker protective response [4]. Our results therefore support the view that a serious injury in older age does not require a fall from a substantial height. Everyday falls occurring while walking, turning, standing up or performing other routine activities may result in a severe fracture.

The mechanism of injury differed by sex, with falls from a height more frequent among men than women. The database did not contain information on the activity at the time of the fall, the location of the fall or the exact height, so it is not possible to explain why this difference arose. Possible differences in exposure, behaviour and activity remain hypotheses that this study cannot test. For a future phase of the project, it would be valuable to record systematically the location of the fall, the activity, the direction of the fall, the use of walking aids, the floor surface and any preceding symptoms.

Cardiovascular disease was the most frequently recorded health condition, present in 78.0% of participants. The systematic review and meta-analysis by Bourke and colleagues confirms that particular cardiovascular disorders are associated with falls in older age and that cardiovascular assessment has a place in risk assessment [6]. In our study, cardiovascular disease was not statistically associated with either fracture site or mechanism of injury. This does not mean that it is unimportant for fall risk, only that within a group of people who had already experienced a fall with fracture it did not differentiate the injury patterns analysed.

Two or more of the four recorded conditions were present in 36.9% of participants. This figure should not be compared directly with the prevalence of multimorbidity in the general population, because our database did not contain a complete list of chronic diseases. A global meta-analysis published in 2025 estimated the prevalence of multimorbidity in older adults at approximately 46%, with very substantial heterogeneity between studies [9]. In our database, having two or more recorded conditions was associated with the mechanism of injury: two or more conditions were more common among people who fell at ground level than among those who fell from a height. This finding may be clinically useful, as it suggests that the overall burden of disease should not be overlooked when assessing a person after a ground-level fall.

Osteoporosis was recorded in 19.2% of participants. It was not associated with fracture site or with hip fracture as a separate outcome, but it was associated with the mechanism of injury: almost 90% of people with recorded osteoporosis sustained their fracture in a ground-level fall. This result should be interpreted with caution. A retrospective database records only a known and documented diagnosis, not systematically assessed bone health. The recent literature warns that osteoporosis may remain insufficiently recognised and documented even after a fragility fracture [12,13]. In one series of adults aged 75 years and over, more than half of the vertebral fractures visible on imaging had not been reported, and those patients went on to sustain more hip and other fractures than patients without vertebral fracture [16]. The absence of recorded osteoporosis in our database should therefore not be equated with confirmed absence of the disease.

At least one previous fracture was recorded in a quarter of participants, but previous fractures were not associated with age, sex, osteoporosis or the number of recorded chronic conditions. This negative result does not contradict the evidence that a previous fracture increases the risk of subsequent fracture. A large meta-analysis used to update the FRAX model showed that a history of fracture significantly increases the risk of subsequent clinical and osteoporotic fracture, including hip fracture [10]. A further meta-analysis demonstrated an increased risk of hip fracture after previous major osteoporotic fractures [11]. Our analysis, however, includes only people who had already sustained an index fracture and does not include data on the site, timing or mechanism of the previous fracture; it was therefore not designed to assess future risk.

The median time from fracture to the start of the recorded rehabilitation episode was 110 days and differed by fracture site and treatment modality. People with a hip fracture had a shorter interval to the start of rehabilitation than people with other fractures, and surgically treated participants had a shorter interval than those treated conservatively. This variable must not be equated with the time to first mobilisation or to early inpatient rehabilitation after surgery. The literature on hip fracture clearly supports early mobilisation and organised rehabilitation care [17,18], but our variable describes the start of the rehabilitation episode recorded in the observed service and not the entire care pathway from acute treatment onwards.

The duration of rehabilitation was highly uniform, with a median of 21 days and an interquartile range of 0 days. This distribution indicates a standardised length of the rehabilitation programme and explains why duration did not differ across most of the characteristics analysed. The weak negative correlation between time to rehabilitation and its duration does not permit a causal interpretation.

The results have direct relevance for nursing practice. World guidelines recommend that, in people at increased risk, assessment should cover previous falls, gait and balance, muscle strength, medication, cardiovascular and neurocognitive factors, functional ability and the environment [2]. Our data show that ground-level falls are most frequent precisely among the oldest people and those with hip fracture, that is, in groups in which the consequences may be particularly severe. Nursing assessment after a fall and during rehabilitation is therefore an opportunity to identify modifiable circumstances, to educate the person and their family, and to refer for further assessment of bone health, medication, balance and home safety. Evidence syntheses indicate that exercise interventions have the most consistent effect on reducing falls and fall-related injuries, which supports the integration of structured exercise into post-fracture rehabilitation and discharge planning [14].

Preventive algorithms should not be built solely on diagnoses available in existing documentation. Future data collection should cover at least fall history, the exact circumstances and location of the fall, functional status, gait and balance, use of walking aids, polypharmacy and fall-risk-increasing medication, orthostatic hypotension, vision and hearing impairment, fear of falling, footwear and environmental hazards. A broader comorbidity profile, including conditions such as chronic kidney disease and hyperlipidaemia, should also be collected systematically. Such a dataset would be closer to the domains recommended by international guidelines [2] and would allow a prospective assessment of their association with new falls.

### 4.1. Strengths and Limitations

The main strength of the study is the analysis of real clinical data from adults aged 65 years and over with clearly documented fractures, including information on the mechanism of injury, chronic conditions, previous fractures, treatment and rehabilitation. A further value is that the data were collected in a Croatian health care setting, for which relatively few studies of post-fall injury patterns have been published.

The study also has several limitations. The retrospective design restricts the analysis to data available in the documentation. There is no comparison group of people who did not fall, so the results cannot be interpreted as predictors of falling. Age was available only in categories rather than as an exact value. The database contained only four groups of chronic conditions, which makes it impossible to assess overall multimorbidity; hyperlipidaemia and chronic kidney disease were not systematically recorded and could not be analysed. Data were not available on medication or polypharmacy, pre-fracture functional status, frailty, gait and balance, orthostatic hypotension, vision or hearing impairment, the location and activity at the time of the fall, the direction of the fall, the use of walking aids, footwear, environmental hazards or bone mineral density. Osteoporosis was based on a recorded diagnosis. The very small number of people rehabilitated on an outpatient basis precluded any reliable comparison of the type of rehabilitation. The analyses were bivariate, and no multivariable modelling was performed; associations reported here are therefore unadjusted. Because multiple exploratory bivariate comparisons were performed without adjustment for multiplicity, individual *p*-values should be interpreted with appropriate caution. No a priori sample size calculation was performed because the cohort consisted of all consecutive eligible patients treated during a fixed retrospective period, and some subgroup comparisons may therefore have had limited power to detect smaller associations. In addition, the Minerva facility was unavailable during part of the study period because of reconstruction and renovation, temporarily reducing the hospital’s rehabilitation capacity and potentially affecting the number and case mix of patients admitted during the study period. Finally, the results describe a population admitted to a rehabilitation programme at a single specialised hospital and may not be generalisable to all older adults who sustain a fracture.

### 4.2. Implications for Nursing Practice and the UNIN Fall Protect Project

The results support directing preventive activities towards ground-level falls, particularly in people of very advanced age. After a fracture, nursing assessment should cover not only the consequence of the fall but also the circumstances in which it occurred, previous falls and fractures, chronic disease, medication, cognitive and functional status, and the conditions for safe mobility.

The finding that osteoporosis was recorded in fewer than one in five participants, despite this being a population with fractures in older age, further supports the need for a clear pathway for the assessment of bone health and for secondary fracture prevention. Nursing interventions may include verifying whether such an assessment has been carried out, providing education on the prevention of further falls and fractures, and coordinating care with the physician, physiotherapist and other team members.

For the next phase of the UNIN Fall Protect project, prospective collection of standardised data that were not available in this retrospective database is recommended. Only such a phase, with follow-up of new falls over time and an appropriate comparison population, can support the development and validation of a fall risk assessment algorithm.

## 5. Conclusions

Among adults aged 65 years and over admitted to a rehabilitation program after a fracture, hip fracture was the most frequent injury and almost four fifths of all fractures followed a ground-level fall. Ground-level falls were particularly frequent among people aged 85 years and over and among those with hip fracture. Most participants had at least one of the recorded chronic conditions, and having two or more recorded conditions, as well as recorded osteoporosis, was associated with the mechanism of injury.

The results do not allow the assessment of causes or the prediction of falls, but they clearly describe a group of older adults in whom a fall ended in fracture. For nursing practice, the need for comprehensive assessment after a fall is particularly important, covering the circumstances of the event, functional and cognitive status, chronic disease, medication, bone health and previous fractures. This approach can serve as a basis for further preventive activities and for the prospective phase of the UNIN Fall Protect project.

## Figures and Tables

**Table 1:** Baseline characteristics of participants (*N* = 255).

Characteristic	Category	*n*	%
Sex	Women	191	74.9
	Men	64	25.1
Age group	65–74 years	95	37.3
	75–84 years	118	46.3
	≥85 years	42	16.5
Fracture site	Hip	135	52.9
	Lower leg	38	14.9
	Femur, excluding hip	35	13.7
	Spine	33	12.9
	Pelvis	14	5.5
Mechanism of injury	Ground-level fall	201	78.8
	Fall from a height	54	21.2
Treatment	Surgical	228	89.4
	Conservative	27	10.6
Type of rehabilitation	Inpatient	253	99.2
	Outpatient	2	0.8

**Table 2:** Mechanism of injury according to selected characteristics.

Characteristic	Fall from a Height, *n*	Ground-Level Fall, *n*	*p*
Men	23	41	<0.001
Women	31	160	
65–74 years	28	67	0.005
75–84 years	24	94	
≥85 years	2	40	
Hip	18	117	<0.001
Femur, excluding hip	3	32	
Lower leg	16	22	
Spine	12	21	
Pelvis	5	9	

*p*-values refer to the overall comparison within each characteristic (chi-square test).

**Table 3:** Time to the start and total duration of rehabilitation.

Characteristic	Time to Rehabilitation, Median (Range; IQR), Days	*p*	Duration of Rehabilitation, Median (Range; IQR), Days	*p*
Total	110 (5–672; 94)	–	21 (4–51; 0)	–
65–74 years	115 (5–672; 96)	0.473	21 (10–40; 0)	0.862
75–84 years	104 (10–377; 87)		21 (4–51; 0)	
≥85 years	109 (17–547; 95)		21 (6–29; 4)	
Hip	97 (14–377; 85)	<0.001	21 (4–43; 0)	0.250
Femur, excluding hip	103 (5–672; 91)		21 (11–40; 10)	
Spine	113 (10–547; 117)		21 (14–51; 2)	
Lower leg	150 (59–483; 89)		21 (10–35; 1)	
Pelvis	126 (85–343; 41)		21 (14–25; 2)	
Conservative treatment	148 (63–672; 92)	<0.001	21 (14–38; 0)	0.814
Surgical treatment	104 (5–547; 92)		21 (4–51; 0)	

IQR–interquartile range. The Kruskal–Wallis test was used for age group and fracture site, and the Mann–Whitney U test for treatment modality.

**Table 4:** Recorded chronic conditions and their association with fracture site and mechanism of injury.

Condition	*n*	%	*p* for Fracture Site	*p* for Mechanism of Injury
Cardiovascular disease	199	78.0	0.389	0.126
Diabetes mellitus	66	25.9	0.771	0.082
Osteoporosis	49	19.2	0.792	0.041
Dementia or cognitive impairment	14	5.5	0.483	0.740

Associations were tested using the chi-square test when expected cell frequencies were adequate. For dementia or cognitive impairment, the Fisher–Freeman–Halton exact test was used for fracture site and Fisher’s exact test for mechanism of injury because of sparse cells.

**Table 5:** Selected characteristics according to the presence of hip fracture.

Characteristic	Hip Fracture: Yes (*n* = 135)	Hip Fracture: No (*n* = 120)	*p*
Men	30	34	0.262
Women	105	86	
65–74 years	43	52	0.064
75–84 years	64	54	
≥85 years	28	14	
Osteoporosis: yes	25	24	0.740
Cardiovascular disease: yes	111	88	0.088
Diabetes mellitus: yes	36	30	0.762
Dementia or cognitive impairment: yes	9	5	0.383
Fall from a height	18	36	0.001
Ground-level fall	117	84	
Time to rehabilitation, median (range; IQR), days	97 (14–377; 85)	125 (5–672; 93)	0.001
Duration of rehabilitation, median (range; IQR), days	21 (4–43; 0)	21 (10–51; 0)	0.766

IQR–interquartile range. The chi-square test was used for categorical variables and the Mann–Whitney U test for continuous variables.

## Data Availability

The data presented in this study are not publicly available because they were extracted from the hospital information system of the Special Hospital for Medical Rehabilitation Varaždinske Toplice and are subject to institutional and data protection restrictions. Anonymised aggregate data may be made available from the corresponding author on reasonable request and with the permission of the institution.

## Supplementary Materials

The following supporting information can be downloaded at https://www.mdpi.com/article/10.3390/geriatrics11050139/s1, Table S1. STROBE Checklist. Figure S1. Flow Diagram of Participant Selection.
