# Supplementary material for: Patterns of Falls and Fractures Among Older Adults Undergoing Rehabilitation After Fall-Related Fractures: A Retrospective Study in Croatia
Source: Geriatrics (Basel). 2026 Oct 2;11(5):139. doi: 10.3390/geriatrics11050139 (PMC13704190; doi:10.3390/geriatrics11050139)
Supplement: Supplementary file 1 [file geriatrics-11-00139-s001.zip › geriatrics-4574299-supplementary.pdf]

Table S1. STROBE Checklist.

STROBE Statement — checklist of items that should be included in reports of observational studies. Completed for: “Patterns of Falls and Fractures Among Older Adults Undergoing Rehabilitation After Fall-Related Fractures: A Retrospective Study in Croatia” (cross-sectional analysis of a retrospective clinical cohort).

Item	Recommendation	Reported in section
1a. Title and abstract	Indicate the study design in the title or abstract	Title; Abstract
1b. Title and abstract	Provide an informative and balanced summary in the abstract	Abstract
2. Background/rationale	Explain the scientific background and rationale	1. Introduction
3. Objectives	State specific objectives, including any prespecified hypotheses	1.1. Aim of the Study
4. Study design	Present key elements of study design early in the paper	2.1. Study Design and Setting
5. Setting	Describe the setting, locations and relevant dates	2.1. Study Design and Setting
6a. Participants	Give eligibility criteria and sources and methods of selection	2.2. Participants
7. Variables	Define all outcomes, exposures, predictors, potential confounders and effect modifiers	2.3. Variables
8. Data sources/measurement	For each variable, give sources of data and details of methods of assessment	2.1. Study Design and Setting; 2.3. Variables
9. Bias	Describe any efforts to address potential sources of bias	2.3. Variables; 4.1. Strengths and Limitations
10. Study size	Explain how the study size was arrived at	2.2. Participants; Figure S1
11. Quantitative variables	Explain how quantitative variables were handled in the analyses	2.2. Participants; 2.4. Statistical Analysis
12a. Statistical methods	Describe all statistical methods, including those used to control for confounding	2.4. Statistical Analysis
12b. Statistical methods	Describe any methods used to examine subgroups and interactions	2.4. Statistical Analysis; 3.6
12c. Statistical methods	Explain how missing data were addressed	2.3. Variables (previous fractures available for 254 participants)
12d. Statistical methods	If applicable, describe analytical methods taking account of sampling strategy	Not applicable
12e. Statistical methods	Describe any sensitivity analyses	Not applicable
13a–c. Participants	Report numbers of individuals at each stage, reasons for non-participation, consider a flow diagram	2.2. Participants; Figure S1

Item	Recommendation	Reported in section
14a. Descriptive data	Give characteristics of study participants and information on exposures and potential confounders	3.1; Table 1
14b. Descriptive data	Indicate number of participants with missing data for each variable of interest	2.3. Variables; 3.5
15. Outcome data	Report numbers of outcome events or summary measures	3.1–3.6; Tables 1–5
16a. Main results	Give unadjusted estimates and, if applicable, confounder-adjusted estimates and their precision	3.1–3.6; Tables 1–5 (bivariate analyses only; see 4.1)
16b. Main results	Report category boundaries when continuous variables were categorized	2.2. Participants; 2.3. Variables
16c. Main results	If relevant, consider translating estimates of relative risk into absolute risk	Not applicable
17. Other analyses	Report other analyses done, e.g. subgroup and sensitivity analyses	3.6. Characteristics of Participants with Hip Fracture
18. Key results	Summarise key results with reference to study objectives	4. Discussion, first paragraph; 5. Conclusions
19. Limitations	Discuss limitations, taking into account sources of potential bias or imprecision	4.1. Strengths and Limitations
20. Interpretation	Give a cautious overall interpretation of results	4. Discussion; 5. Conclusions
21. Generalisability	Discuss the generalisability of the study results	4.1. Strengths and Limitations
22. Funding	Give the source of funding and the role of the funders	Funding; Conflicts of Interest

Reference: von Elm E, Altman DG, Egger M, Pocock SJ, Gøtzsche PC, Vandenbroucke JP. The Strengthening the Reporting of Observational Studies in Epidemiology (STROBE) statement: guidelines for reporting observational studies. *Lancet* 2007;370:1453–1457. Checklist available at https://www.strobe-statement.org

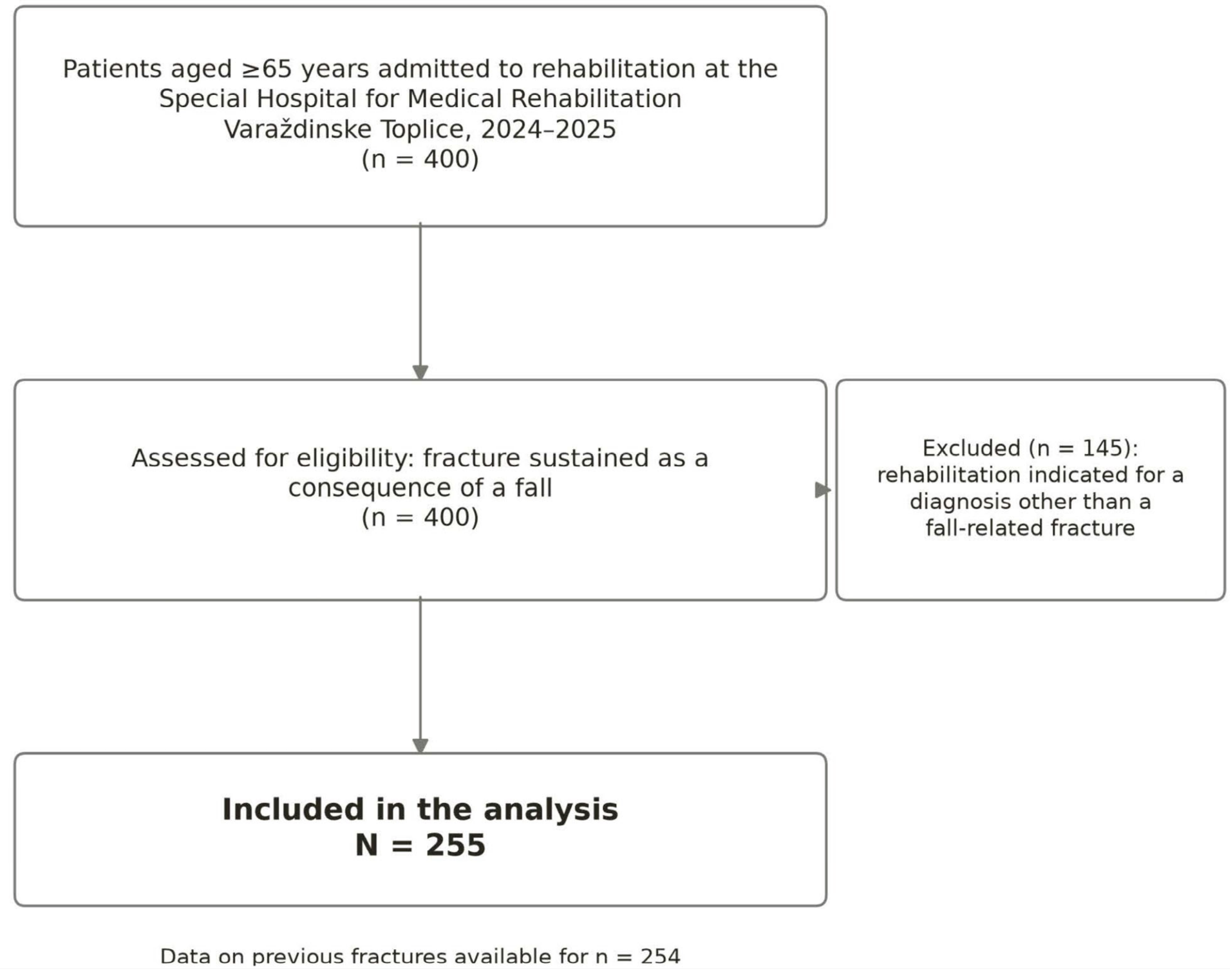

Figure S1. Flow diagram of participant selection.
